# Prevalence, Time of Infection, and Diversity of Porcine Reproductive and Respiratory Syndrome Virus in China

**DOI:** 10.3390/v16050774

**Published:** 2024-05-13

**Authors:** Chaosi Li, Aihua Fan, Zhicheng Liu, Gang Wang, Lei Zhou, Hongliang Zhang, Lv Huang, Jianfeng Zhang, Zhendong Zhang, Yan Zhang

**Affiliations:** 1 Boehringer Ingelheim Animal Health (Shanghai) Co., Ltd., Shanghai 200040, China; lichaosi14@163.com (C.L.); lv.huang@boehringer-ingelheim.com (L.H.); 2 Key Laboratory of Livestock Disease Prevention of Guangdong Province, Institute of Animal Health, Guangdong Academy of Agricultural Sciences, Guangzhou 510640, China; rainman136@aliyun.com (Z.L.); 13668939298@139.com (J.Z.); 3 College of Veterinary Medicine, Inner Mongolia Agricultural University, Hohhot 010010, China; 4 College of Veterinary Medicine, Shandong Agricultural University, Tai’an 271000, China; wg0381@163.com; 5 National Key Laboratory of Veterinary Public Health Safety, College of Veterinary Medicine, China Agricultural University, Beijing 100094, China; leosj@cau.edu.cn; 6 Key Laboratory of Animal Epidemiology of Ministry of Agriculture and Rural Affairs, College of Veterinary Medicine, China Agricultural University, Beijing 100094, China; 7 State Key Laboratory for Animal Disease Control and Prevention, Harbin Veterinary Research Institute, Chinese Academy of Agricultural Sciences, Harbin 150001, China; zhanghongliang01@caas.cn; 8 School of Biotechnology, Jiangsu University of Science and Technology, Zhenjiang 212000, China; zhangzhend90@126.com; 9 Branch of Animal Husbandry and Veterinary of Heilongjiang Academy of Agricultural Sciences, Qiqihar 161006, China; zhangyanyan008@163.com

**Keywords:** PRRSV, prevalence, three-sample strategy, time of infection, wild strain diversity

## Abstract

Porcine reproductive and respiratory syndrome virus (PRRVS) is a major swine viral pathogen that affects the pig industry worldwide. Control of early PRRSV infection is essential, and different types of PRRSV-positive samples can reflect the time point of PRRSV infection. This study aims to investigate the epidemiological characteristics of PRRSV in China from Q4 2021 to Q4 2022, which will be beneficial for porcine reproductive and respiratory syndrome virus (PRRSV)control in the swine production industry in the future. A total of 7518 samples (of processing fluid, weaning serum, and oral fluid) were collected from 100 intensive pig farms in 21 provinces, which covered all five pig production regions in China, on a quarterly basis starting from the fourth quarter of 2021 and ending on the fourth quarter of 2022. Independent of sample type, 32.1% (2416/7518) of the total samples were PCR-positive for PRRSV, including 73.6% (1780/2416) samples that were positive for wild PRRSV, and the remaining were positive for PRRSV vaccine strains. On the basis of the time of infection, 58.9% suckling piglets (processing fluid) and 30.8% weaning piglets (weaning serum) showed PRRSV infection at an early stage (approximately 90% of the farms). The sequencing analysis results indicate a wide range of diverse PRRSV wild strains in China, with lineage 1 as the dominant strain. Our study clearly demonstrates the prevalence, infection stage, and diversity of PRRSV in China. This study provides useful data for the epidemiological understanding of PRRSV, which can contribute to the strategic and systematic prevention and control of PRRSV in China.

## 1. Introduction

Porcine reproductive and respiratory syndrome (PRRS) is one of the most critical viral swine diseases caused by porcine reproductive and respiratory syndrome virus (PRRSV), which is an enveloped, positive-sense, and single-stranded RNA virus belonging to the order Nidovirales and family Arteriviridae [1]. PRRSV infection causes clinical symptoms such as immunosuppression, reproductive disorders in pregnant sows, and respiratory diseases in piglets, thereby threatening the pig industry worldwide [2,3]. The PRRSV genome is approximately 15,000 nucleotides in length with a 5′-untranslated region and a poly(A) tail at the 3′-terminus. It consists of at least 11 open reading frames (ORFs), e.g., ORF1a, ORF1b, ORF2a, ORF2b, ORF3-7, ORF5a, and ORF2TF [4,5]. Within the PRRSV genome, ORF5 encodes glycoprotein 5, which has key roles in different biological processes such as the virus entry into target cells and activation of the host immune response [2,6].

PRRS was reported for the first time in North Carolina in 1987. The PRRSV strains were classified as PRRSV-1 and PRRSV-2 (Betaarterivirus suid 1 and Betaarterivirus suid 2, respectively), which share approximately a 60% nucleotide sequence homology [7].

In China, both PRRSV-1 and PRRSV-2 have been reported, and they cause economic losses for the swine-rearing industry [8]. PRRSV-1 has been detected in at least 23 regions in China, and all of them were subtype 1 [8,9]. According to the genetic characteristics of ORF5, PRRSV-2 strains have been divided into 9 lineages and 37 subfamilies [10,11]. In 2006, outbreaks caused by a highly pathogenic PRRSV (HP-PRRSV) were found in swine herds, which displayed high fever and severe reproductive disorders [12]. In 2012, the spread of a new strain type, NADC30, drew widespread attention in China [13,14]. Currently, lineage 1, lineage 3, and lineage 8 are the most prevalent strains of PRRSV-2 [15]. From 2017 to 2019, phylogenetic analyses based on the ORF5 gene revealed that the detection rates of lineage 1, lineage 3, and lineage 8.7 were 62.9% (39/62), 21% (13/62), and 14.5% (9/62), respectively [16]. An increased recombinant frequency of PRRSV strains has led to a progressively complex epidemic situation in China, and it poses considerable challenges for the prevention and control of PRRS [17,18].

The detection rate of PRRSV varies depending on location in China. In South China, 6795 clinical samples from diseased pigs were collected from 2017 to 2021, and 18.82% of them were positive on the basis of PRRSV real-time (RT) polymerase chain reaction(PCR) [2]. In East China, 231 samples (lung or serum) were collected from 2017 to 2022, and 24% (54/231) of the samples were positive for PRRSV [19]. In Shanxi Province, 491 pigs from 19 slaughterhouses were sampled in 2019, and the positive results for PRRSV PCR were 11.82% [20]. In Shandong Province, of the 637 tissue samples collected from June 2018 to June 2019, 9.58% were positive for PRRSV [21]. In Hunan and Hebei Provinces, 482 out of 5799 samples (8.31%) were positive for PRRSV via RT-PCR in 2021 [22].

Since the outbreak of African swine fever (ASF) in 2019, no epidemiological investigation has been conducted to comprehensively map PRRSV prevalence and dominant strains in all major pig production regions in China. In this study, a total number of 7518 samples (of processing fluid, weaning serum, and oral fluid) were collected from 100 intensive pig farms in 21 provinces, which covered all five pig production regions in the nation, on a quarterly basis from the fourth quarter of 2021 to the fourth quarter of 2022. This study investigated the epidemiological characteristics of PRRSV in China, including various age groups, production regions, different quarters, and time of infection, which would be of benefit for PRRSV strategic control in the nation.

## 2. Materials and Methods

### 2.1. Sampling Strategy

**Figure 1:**
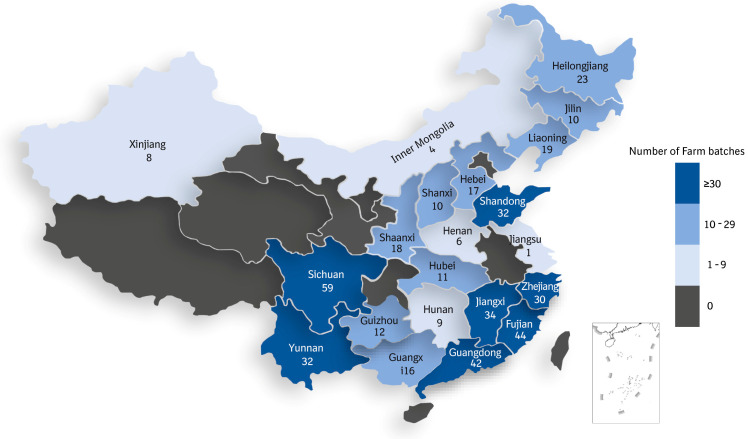
The geographical locations of the farm sample batches.

A cross-sectional study was performed in 100 intensive pig farms (ranging from 500 to 6000 sows) from 21 provinces in China (Figure 1) on a quarterly basis (from the fourth quarter of 2021 to the fourth quarter of 2022). The investigated farms covered all five pig production regions classified by the Ministry of Agriculture and Rural Affairs in China, which include the regions of the north, east, northwest, southwest, and central–south. The north region includes nine provinces (Beijing, Tianjin, Hebei, Shanxi, Inner Mongolia, Liaoning, Jilin, and Heilongjiang). The east region is composed of six provinces (Shanghai, Jiangsu, Zhejiang, Anhui, Shandong, and Henan). Six provinces (Fujian, Jiangxi, Hunan, Guangdong, Guangxi, and Hainan) are included in the central–south region. The southwest region contains Hubei, Chongqing, Sichuan, Guizhou, Yunnan, and Tibet. The northwest region includes Gansu, Qinghai, Ningxia, Shaanxi, and Xinjiang. The distribution of eligible farms was allocated according to the distribution of marketed finishers in 2020. However, there were some deviations because of the severity of the ASF outbreak in central China, as well as due to the impact of the COVID-19 quarantine policy on sample transportation.

The sampling strategy is described in Table 1. Ideally, each batch of samples was requested to include three types of samples, as indicated in Table 1. In terms of the definition of a positive batch, as long as one sample was detected by RT-PCR as PRRSV or wild-PRRSV-positive independent of sample type, the batch was classified as positive for PRRSV or wild PRRSV. To calculate the number wild PRRSV samples, RT-PCR positive samples of each positive batch were performed by sequencing, and the relevant number was taken into account for a positive reading of specific vaccine-like or wild strain-based sequencing results. The samples with a similarity to ORF5 was ≥98% when compared with the commercial vaccine strains in China that were classified as a vaccine-like strain [23]. There is no PRRSV-1 vaccine commercially available in China; therefore, PRRSV-1 positive samples were not taken as the vaccine strain but rather as the wild strain. However, the ASF outbreak and COVID-19 limited the implementation of a planned sampling strategy, thus leading to certain incomplete sample batches. A total of 7518 samples, which included three types of samples (i.e., processing fluid, weaning serum, and oral fluid from 437 batches/farms (one batch per farm), were collected to determine the prevalence of PRRSV. These included 3001 processing fluid samples from 411 batches, 2628 weaning serum samples from 412 batches, and 1889 oral fluid samples from 307 batches, which were as represented 39.9% (3001/7518), 34.9% (2628/7518), and 25.1% (1889/7518) of the total samples, respectively.

The three-sample strategy was used to determine the timing, herds, and/or place of PRRSV infection. On the basis of the three-sample strategy, the timing and/or place of the PRRSV infection was defined when the first test read positive in the following order: processing fluid (i.e., the PRRSV circulation in sows and horizontal transmission among new-born piglets), weaning serum (the early infection in sucking piglets), and oral fluid (the PRRSV infection in nurseries) in each farm per quarter. In total, 263 of 437 batches were selected to determine the timing of the PRRSV infection, of which each batch contained three required types of samples.

### 2.2. Sampling Procedure

The processing fluid collection was implemented during a castration procedure at 3–5-day-old nurseries following the guidelines by Lopez et al. [24]. For the batch-farrowing farms, the processing fluids were obtained at all piglet processing times, one aggregated processing fluid sample per 20–30 litters was collected and stored in a 50 mL centrifuge tube at − 20 °C. Oral fluid collection was conducted during at 8–10-week-old nurseries according to the guidelines by Prickett et al. [25]. Briefly, cotton ropes were positioned at shoulder height for the pigs, and these were hanged within the pen for 20 to 30 min. The pigs were naturally exposed to the rope and oral fluids that were collected during the process of interaction. Oral fluids were collected from the rope into a 50 mL centrifuge tube. The samples were stored at −20 °C. Blood samples from 30 weaning piglets were randomly selected from a batch and collected using a disposable syringe. Blood was centrifuged at 1000× *g* for 10 min, and the serum was harvested and stored at −20 °C. All samples were submitted to the labs after being fully prepared. All pigs were released after sampling.

### 2.3. PCR Detection

RNA was extracted from all 7518 samples by using extraction kits (TianLong Science and Technology Co., Ltd., Xi’An, China) according to the manufacturer’s instructions [26]. Real-time PCR was performed to detect the presence of PRRSV nucleic acids by using a commercial PRRSV RT-PCR detection kit (VetMAX™ PRRSV EU & NA 2.0 Kit; Thermo Fisher Scientific, Waltham, MA, USA) [27]. The sequences with mixed or overlapped signals were implemented into multiplex RT-PCR for the differential detection of lineage 8.7, lineage 1.8, and lineage 1.5 PRRSV (PRRSV Lineage8.7&Lineage1.8&Lineage1.5 Kit; GuanMu diagnosis Co., Ltd., Changsha, China).

### 2.4. ORF5 Sequencing and Genetic Analysis

All PCR-positive samples with Ct values less than 30 were collected and sent to Sangon Biotechnology Co., Ltd., Shanghai, China for ORF5 sequencing [28]. The sequence data were assembled and analyzed using Lasergene (DNAstar, Madison, WI, USA) and DNAMAN (Lynnon Biosoft, Vaudreuil-Dorion, QC, Canada). Multiple-sequence alignments were performed using Clustal W.

The evolutionary history of the PRRSV strains was inferred using the Maximum Likelihood method based on the Tamura–Nei model [29]. The bootstrap consensus tree inferred from 1000 replicates [30] was used to represent the evolutionary history of the analyzed taxa [30]. The branches corresponding to partitions reproduced in less than 50% bootstrap replicates were collapsed. The initial trees for the heuristic search were obtained automatically by applying the neighbor joining and BioNJ algorithms to a matrix of pairwise distances, which were estimated using the Maximum Composite Likelihood approach, and the topology with the superior log likelihood value was then selected. Codon positions were 1st + 2nd + 3rd + Noncoding. All positions containing gaps and missing data were eliminated. Evolutionary analyses were conducted using MEGA5 [31]. A total of 390 ORF5 genes of mono-PRRSV infection-positive samples were taken into the phylogenetic analysis.

Five hundred PRRSV-positive samples were considered for the prevalence proportion analysis, including 390 mono-PRRSV infection-positive samples, 75 PRRSV-1 positive samples confirmed by RT-PCR, 25 PRRSV-1&PRRSV-2 co-infection samples, and 10 multi-strain samples, and these were defined by multiplex RT-PCR. In the meantime, the 500 samples that came from 170 batches were investigated via prevalence proportion analysis based on batches and ages.

Twenty ORF5 genome sequences, including lineage 1.5, lineage 1.8, lineage 3, and lineage 8, were detected in this study, which were then selected and submitted to GenBank; the accession numbers are listed in Table 2. Information on the rest of the ORF5 sequences is available in Supplementary Table S1.

## 3. Results

### 3.1. PRRSV Prevalence and Distribution in China

#### 3.1.1. At the National Level

**Figure 2:**
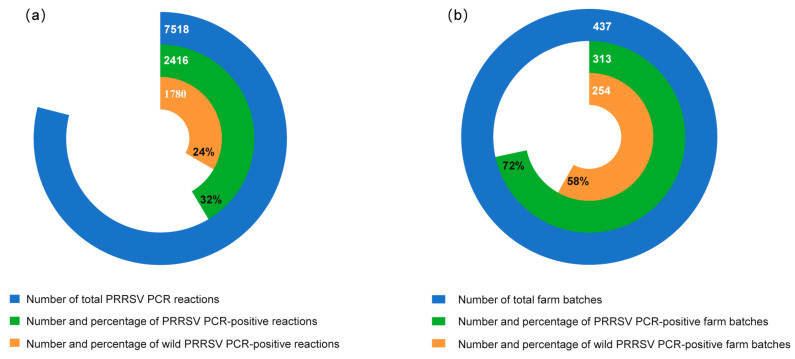
Results of Porcine reproductive and respiratory syndrome virus (PRRSV) polymerase chain reaction (PCR)s from Q4 2021 to Q4 2022. (**a**) Number and percentage of total, PRRSV PCR-positive, and wild PRRSV PCR-positive reactions. (**b**) Number and percentage of total, PRRSV PCR-positive, and wild PRRSV PCR-positive farm batches.

A total of 7518 samples were collected to determine the prevalence of PRRSV at farm and regional levels. Independent of sample type, 2416 samples were PRRSV-positive, which accounted for 32.1% (2416/7518) of the total samples (Figure 2a). Among the PRRSV-positive samples, 73.6% (1780/2416) samples were specifically positive for wild PRRSV strains (up to 23.7% of total samples). The remaining 636 positive samples were classified as PRRSV vaccine-like strains, including 7.7% (582/7518) respPRRS MLV (modified live vaccine)-like and 0.7% (54/7518) TJM-F92-vaccine-like samples. The rate of the PRRSV-positive farm batches was 71.6% (313/437) and the average rate of wild PRRSV-positive farm batches was 58.1% (254/437), thereby indicating a high prevalence of PRRSV across different regions (Figure 2b).

#### 3.1.2. At the Regional Level

**Figure 3:**
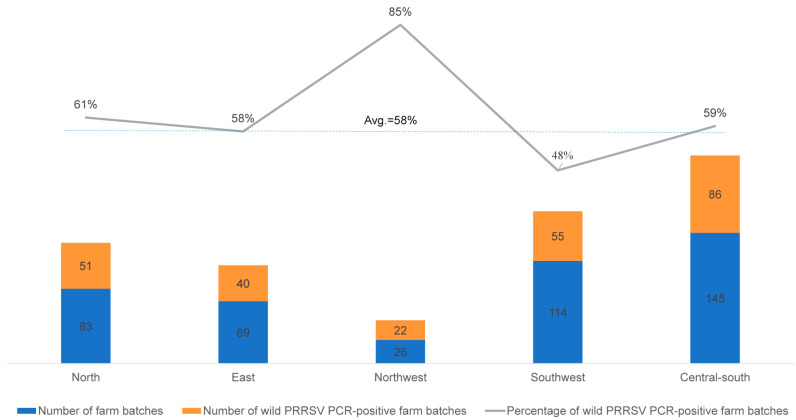
Number and percentage of wild PRRSV PCR-positive farm batch-based samples in each of the five production regions.

The field PRRSV-positive rates in the regions of the north, east, and central–south (61.4%, 57.9%, and 59.3%, respectively) were similar to the average. The northwest region was found to have the highest positive rate at 84.6% (22/26), and the southwest region was detected as the lowest rate at 48.2% (55/114) (Figure 3).

#### 3.1.3. At the Age and Quarterly Levels

**Figure 4:**
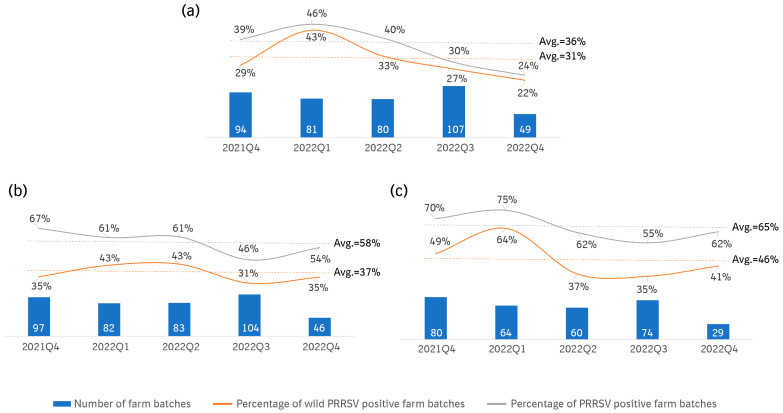
(**a**) The total/wild PRRSV PCR-positive rates of the processing fluid samples in each quarter. (**b**) The total/wild PRRSV PCR-positive rates of the weaning serum samples in each quarter. (**c**) The total/wild PRRSV PCR-positive rates of the oral fluid samples from 8–10-week-old piglets in each quarter.

The PRRSV- and wild PRRSV-positive rates of each sample type in different quarters by batches are presented in Figure 4a–c. Both the total and wild PRRSV-positive rates showed similar trends, where higher detection rates were observed in the older piglets. The average total PRRSV-positive rates in the 3–5-day-old suckling pigs, 3–4-week-old weaning pigs, and the 8–10-week nurseries were 36%, 58%, and 65%, respectively, and the wild PRRSV-positive rates were 31%, 37%, and 46%, respectively. All three sample types showed similar trends of wild PRRSV-positive rates. The peak was detected in the first quarter of 2022, with the highest average in the oral fluid samples (64%), and 43% average positive rates for the processing fluid and weaning serum samples. The lowest average positive rate was observed in the third quarter of 2022 for both the weaning serum and oral fluid samples, with the exception of the processing fluid samples (which were found in a later quarter).

#### 3.1.4. At the Provincial Level

The detection rate of total/wild-type PRRSV varied among provinces (Table 3 and Table 4). Based on the samples, the first three provinces with the highest PRRSV-positive rate were Hebei, Yunnan, and Shandong (59.0%, 53.1%, and 52.1%, respectively). The three provinces with the highest detection rates for wild PRRSV were Hebei, Heilongjiang, and Shandong (44.8%, 42.2%, and 39.4%, respectively), and the detection rates were higher than the average of 23.7%. The three provinces with the lowest positive reaction rates were Guizhou, Hunan, and Jilin (0.7%, 2.8%, and 6.6%, respectively), but the total number of PCRs in these provinces were less than the average number of PCRs. Shaanxi, Fujian, and Guangdong Provinces had the first three largest number of samples (781, 852, and 1044 number of samples, respectively), and the detection rates for wild PRRSV strains were 31.5%, 20.0%, and 22.5%, respectively.

Based on the batches, the first three provinces with the highest detection rate were Jiangsu, Henan, and Shandong (100.0%, 100.0%, and 96.9%). The three provinces with the highest detection rates for wild PRRSV were Jiangsu, Shaanxi, and Hebei (100.0%, 94.4%, and 88.2%, respectively). But the total number of batches in Jiangsu Province (1 batch) in terms of statistics was much less than the average.

### 3.2. The Three-Sample Strategy Was Used to Determine the Time of the PRRSV Infection

**Figure 5:**
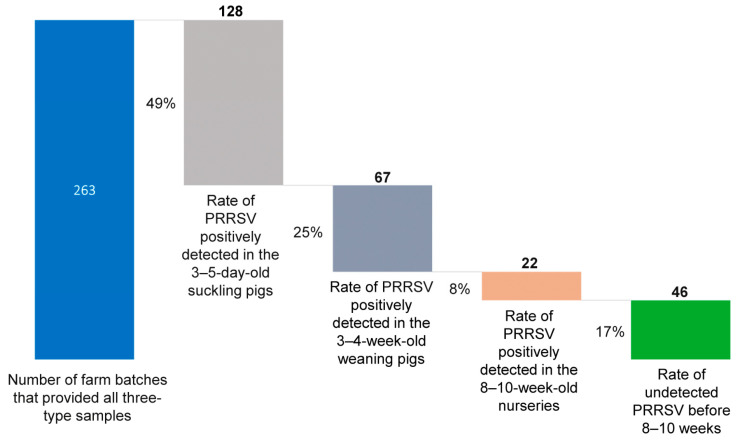
The timing of PRRSV infection determined using the three-sample strategy.

Out of the 437 farm batches, 263 contained three types of samples during each sampling. As indicated in Figure 5, 17.5% (46/263) of the farm batches showed negative results for PRRSV before the late nursery stage, 48.7% (128/263) of the farm batches showed positive results for PRRSV infection and/or circulation in the 3–5-day-old piglets, 25.5% (67/263) of the farm batches showed early infection in weaning piglets, and the remaining 8.4% (22/263) showed PRRSV infection in the nurseries. Among all the 217 PRRSV-positive farm batches, nearly 89.8% (195/217) were identified to have early infections in either the neonatal or sucking piglets, which may have been vertically and/or horizontally transmitted from the sows.

### 3.3. Sequencing-Analysis-Revealed PRRSV Wild Strain Diversity

**Figure 6:**
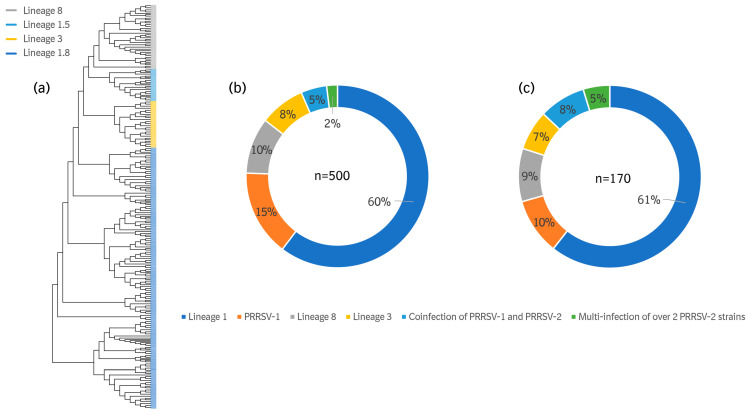
(**a**) Phylogenetic analysis of the 390 ORF5 mono-PRRSV-2 infection-positive samples in China during Q4 2021 to Q4 2022. (**b**) Prevalence rate of the PRRSV strains by samples. (**c**) Prevalence rate of the PRRSV strains by farm batches.

The results indicate the complexity of the PRRSV circulation, including both PRRSV-1 and PRRSV-2, in China. In the samples, 5% were detected as holding a co-infection of PRRSV-1 and PRRSV-2, and multi-PRRSV-2 strain infections accounted for up to 2% of the total (Figure 6b).

To understand the PRRSV-2 lineages circulating in China, 390 ORF5 genes of mono-PRRSV infection-positive samples were sequenced before the phylogenetic analysis (Figure 6a). Of these, lineage 1, lineage 3, and lineage 8 of PRRSV-2 were detected with 300, 40, and 50 ORF5 sequences, which accounted for 60%, 8%, and 10%, respectively, from a total of 500 samples. Within lineage 1, lineage 1.5 and lineage 1.8 accounted for 5.4% and 54.6%, respectively, out of a total of 500 samples (Figure 6a).

For sequencing, the samples collected from 170 farm batches were also considered. Multi-PRRSV-2 strain infections (5%) and a co-infection of PRRSV-1 and PRRSV-2 (8%) were observed to be higher than those considered in the sample number-based analysis. The proportions of lineage 1, lineage 3, and lineage 8 of PRRSV-2 demonstrated similar trends, as shown in Figure 6b,c.

PRRSV-1 and PRRSV-2 both included each lineage distribution in the three age classes, as presented in Supplementary Table S2.

## 4. Discussion

PRRS, which is caused by PRRSV, has been considered as one of the most critical swine diseases threatening the swine industry in China and worldwide [2,32,33,34]. The genetic diversity and prevalence of PRRSV in China have been reported in numerous studies throughout the years. The prevalence of HP-PRRSV in 2006 caused economic losses [12], and it was a major epidemic strain of PRRSV that circulated in China [35]. Recently, the prevalence rates of other PRRSV lineages, particularly NADC30-like strains [36,37] and NADC34-like strains [38], have increased and have gradually become dominant. Therefore, epidemiological investigations of intensive pig farms at the national level are essential for understanding the characteristics of PRRS. However, most of the previous studies had limitations regarding either the number of investigated provinces and/or the regions or farm scales. Therefore, this study was designed to investigate the prevalence, time of infection, and genetic diversity of PRRSV via a three-sample and active sampling strategy in 21 provinces of China, which covered all five pig production regions.

In this study, wild PRRSV-positive samples accounted for 73.6% (1780/2416) of the PRRSV-positive samples, which, in turn, accounted for up to 23.7% of the total samples. The positive rate of the samples in this study was found to be higher than in previous studies (8.31–18.82%) even though most of them were clinical samples [2,21,22]. This may be due to the following three reasons: Firstly, the specific background of this period, i.e., where most of farms had repopulated and expanded rapidly due to the positive expectations in the market for 2022; as such, there was not enough gilt supplement because of the ASF that had spilled out, and farms had to introduce the gilts from several sources without a sufficient acclimation period. Secondly, most of the previous epidemiological studies used individual samples such as serum, swab, or tissue, while this study used population-based samples, which have a greater possibility to sample PRRSV-positive piglets [39]. This study included 21 provinces from 5 production regions, which covered more terrain and climate variations than has been captured in previous studies in more restricted areas. Lastly, but not least, all of the samples in this study were tested by commercial kits, which may have more sensitivities [27,40].

Compared to the detection rate of the wild PRRSV samples at 23.7%, the rate of vaccine-like samples was much lower at 8.4% (636/7518). The difference in detection rates may be related to the suspension or reduction in the frequency of vaccine immunization in farms during the susceptible and precise elimination period of ASF. Meanwhile, the PRRS MLV vaccination was generally performed at 2–3 weeks of age, and vaccinemia lasted for about 5–8 weeks and turned to negative at 7–11 weeks [41,42]. In this study, 39.9% (3001/7518) of the samples were processing fluid (3–5-day-old piglets), and 25.1% (1889/7518) of them were oral fluid from 8–10-week-old nursery piglets, so the likelihood of detecting the vaccine-like strain is relatively low. Among the 8.4% vaccine-like samples, 91.5% (582/636) were respPRRS MLV-like samples and 8.5% (54/636) were TJM-F92-vaccine-like samples, which may be related to respPRRS MLV and TJM-F92-vaccine-like strains, which have a higher market share in China.

However, an interesting observation in this study was that 58.1% (254/437) of farm batches were positive for wild PRRSV, which is higher than that at the individual sample level (23.7%, 1780/7518). On average, 7.0 (1780/254) wild PRRSV-positive samples were detected in each wild PRRSV-positive batch, and the average number of submitted samples per batch was 17.2 (7518/437). Therefore, in each wild PRRSV batch, the average proportion of wild PRRSV sample was 40.7% (7.0/17.2). These data indicate that, before pigs are 8–10-weeks-old, 40.7% of piglets will be infected by wild PRRSV in 58.1% (254/437) of batches.

The detection rate of the wild PRRSV batches varied among the 5 reproduction regions and 21 provinces. The regions that had an above average wild PRRSV-positive batches rate were the northwest, north, and central–south. The southwest was the only region below the average level. Since the number of batches in the northwest region was only 26 (i.e., lower than the median of 83 batches and the average of 87.4 batches), the results may be biased due to an insufficient sample size. Compared to the southwestern region, the north region had a higher average altitude, lower yearly average temperature, and lower relative humidity, which might be part of the reasons for the higher number of positive batches. Specific to each province in the north, such as Hebei and Heilongjiang, there were much higher detection rates than the average (24%) and the batches (58%) in the samples. The reasons for this may not only include climate, but also the terrain characteristics of areas where a longer duration of an outdoor temperature of <4 °C was observed. It may lead to difficulties in PRRSV disinfection in daily production practices, in external biosecurity execution, as well as in the balance of a sufficient ventilation and maintenance of a comfortable indoor temperature. Arruda et al. reported that the influence of terrain characteristics on the spread of airborne diseases, such as PRRS [43]. Jara M. et al. also demonstrated that a higher elevation will be of more benefit for PRRSV control [44]. Based on these studies, it can be speculated the north region shows higher PRRSV prevalence as most of the areas in the northern part of China are plains and have a lower attitude than the average level, especially when compared with the northwest.

A previous study [45] reported that the PRRSV detection in different samples provides evidence for infection timing determination and breeding farm classification, which can contribute to appropriate control actions and/or strategies. The aforementioned study also reported an increased number of born-alive piglets and a reduction in piglet pre-weaning mortality in a breeding farm where PRRSV stability was achieved and maintained for a year [45]. Trevisan et al. [46] assessed the economic impacts of PRRSV infection and demonstrated the relevance between early infection and economic losses. The results of this study, however, showed that 89.8% (195/217) of the investigated farm batches were found to have early wild PRRSV infection in 3–5-day-old sucking piglets (58.9%, 128/217) and weaning piglets (30.9%, 67/217). This means that most of the investigated farms were categorized as positively unstable on the basis of the classification of swine herds by PRRSV status.

The data of the timing of infection provide a good reference for vaccine immunization strategy improvement, and they also partially explain why producers have always complained about the PRRSV vaccine’s insufficient efficacy. Optimizing the vaccination timing of piglets firstly requires a determination of the time of PRRSV wild virus infection. The efficacy onset after vaccination needs at least >28 days [5]. Implementing MLV immunization should be conducted 4 weeks before a wild PRRSV infection can take root, and this will result in a sufficient clinically protective performance. However, as inferred from the results discussed above, 74% of the batches showed that wild PRRSV infection occurred before weaning (3–4 weeks-old). Most farms in China implemented a fixed PRRS vaccination scheme for 2–3-week-old piglets, which resulted in that vaccination not having the desired onset of efficacy before the wild PRRSV infection occurred in the herd. Therefore, we kindly suggest that farms should keep monitoring and tracing the infection time of the PRRSV wild virus in their herds, and they should continuously improve their PRRSV vaccination strategy by determining the timing of infection. If the majority of the piglets have been infected before weaning, the priority is to stabilize the sow herd by herd closure and mass vaccination twice, 4 weeks apart.

Sequence analysis of the ORF5 gene in the 390 PRRSV strains were performed in this study. The results revealed the complexity and diversity of PRRSV prevalence in China, which is attributable to numerous drivers that include, but are not limited to, raising mixed sources of breeding pigs, resuming production and replacement after the ASF outbreak in 2019, the needs of the farm and profit-driven cross-region transportation, and the use of different PRRSV vaccine strains in the same farm. Consistent with the findings of previous studies [8,9,22], the results in Figure 6 show that wild PRRSV strains in China include both PRRSV-1 and PRRSV-2, which comprise lineage 1, lineage 3, lineage 5, and lineage 8. Lineage 5 was not presented in Figure 6 as it is considered a PRRS vaccine strain in China. Lineage 1 was shown as the dominant strain, accounting for 60% in the samples and 61% in the farm batches. The rate of PRRSV-1 at the sample and farm batch levels was 15% and 10%, respectively, which was higher than the rate of lineage 8 (9% and 8%, respectively). A relatively low pathogenicity and no commercial PRRSV-1 strain vaccine in China may be the root causes for its high prevalence. Interestingly, a recent study indicated that the PRRSV-2 strain vaccine may provide efficacious protection against the challenge by the PRRSV-1 strain [47]. This may be considered as an alternative option for PRRSV-1 prevention and control in China in the absence of a commercial PRRSV-1 strain vaccine. In our study, the co-infection of PRRSV-1 and PRRSV-2 together with a multi-infection of PRRSV-2 strains accounted for 7% at the sample level and 13% at the farm batch level. Breeding herd production performance is associated with the number of PRRSV strains circulating in farms [28]. The investigation of this study indicated that more than three PRRSV strains were detected in farms, and that they can cause 1827 fewer piglet losses per 1000 sows. When converting from external gilt introduction to self-breeding, the improvement of external biosecurity, an appropriate vaccination strategy, and an optimized PRRS vaccine selection criteria may be considered as effective approaches to reduce co-infection and multi-infection at the farm level.

In conclusion, we investigated the prevalence in the nation via obtaining samples from 5 production regions and 21 provinces. We recorded the time of infection and diversity of PRRSV in China. The results of this study clearly demonstrate the high prevalence of PRRSV at the farm level, the high early PRRSV infection rate, and it offers comprehensive information on PRRSV diversity. The findings provide useful data and insights that can improve our understanding from an epidemiological perspective, and it may contribute to the prevention and control of PRRSV in China.

## 5. Conclusions

We investigated the prevalence, time of infection, and diversity of PRRSV in China. The results of this study clearly demonstrate the higher prevalence of PRRSV, especially in the north region, than other relative reports at both the sample and batch levels. The results from the three age stages demonstrate that a higher prevalence of wild PRRS in batches was found in older age groups. They also confirm a high early PRRSV infection rate and offer comprehensive information on PRRSV diversity. The findings provide epidemiological data at the national level and may contribute to the prevention and control of PRRSV in China.

## Figures and Tables

**Table 1:** Sampling strategy.

Type of Sample	Age of Piglet	Sample Size	Pattern of Sampling	Type of Test
Processing fluid	3–5 days	Collected from all male piglets per batch, and the number varied within the farms and batches based on the sow inventory number	Pools of 20–30 litters	Real-time PCR
Weaning serum	3–4 weeks before weaning	30 samples/batch	Fixed spatial sampling with no more than one sample per litter with pools of 5	Real-time PCR
Oral fluid	8–10 weeks in nurseries	6 samples/batch	Fixed spatial sampling	Real-time PCR

**Table 2:** Porcine reproductive and respiratory syndrome virus (PRRSV) open reading frames 5 (ORF5) sequence codes and GenBank accession numbers.

Strain	Accession No.	Genotype	Province
FJSM	PP315334	Lineage 8	Fujian
GDFS	PP315335	Lineage 3	Guangdong
GDHY-2	PP315336	Lineage 3	Guangdong
YNQJ	PP315337	Lineage 1.8	Yunnan
SXWN-4	PP315338	Lineage 1.8	Shaanxi
SCSN	PP315339	Lineage 1.8	Sichuan
SXWN-2	PP315340	Lineage 1.8	Shaanxi
GSTS	PP315341	Lineage 1.8	Shaanxi
FJNP	PP315342	Lineage 1.8	Fujian
GDHZ	PP315343	Lineage 1.8	Guangdong
SCGY-1	PP315344	Lineage 1.8	Sichuan
YNWS	PP315345	Lineage 1.8	Yunnan
SXWN-1	PP315346	Lineage 1.8	Shaanxi
JXJA	PP315347	Lineage 1.5	Jiangxi
JXFZ	PP315348	Lineage 1.5	Jiangxi
SXWN-3	PP315349	Lineage 1.8	Shaanxi
SCGY-2	PP315350	Lineage 1.8	Sichuan
SCMS	PP315352	Lineage 1.8	Sichuan
XJYL	PP315353	Lineage 1.8	Xinjiang
SCDZ	PP315354	Lineage 1.8	Sichuan
respPRRS MLV	AF066183.4	Lineage 5	Reference strain
TJM-F92	MN508255.1	Lineage 8	Reference strain

**Table 3:** The number and percentage of total/wild PRRSV PCR-positive reactions in the 21 provinces.

Province	Production Region	Number of PRRSV PCR-Positive Reactions	Number of Wild PRRSV PCR-Positive Reactions	Number of PRRSV PCRs	Percentage of PRRSV PCR-Positive Reactions	Percentage of Wild PRRSV PCR-Positive Reactions
Guangdong	Central–south	297	235	1044	28.4%	22.5%
Fujian	Central–south	340	170	852	39.9%	20.0%
Jiangxi	Central–south	182	98	524	34.7%	18.7%
Guangxi	Central–south	80	78	245	32.7%	31.8%
Hunan	Central–south	8	3	109	7.3%	2.8%
Zhejiang	East	81	45	475	17.1%	9.5%
Shandong	East	225	170	432	52.1%	39.4%
Henan	East	45	34	121	37.2%	28.1%
Jiangsu	East	14	7	36	38.9%	19.4%
Liaoning	North	29	26	352	8.2%	7.4%
Heilongjiang	North	142	129	306	46.4%	42.2%
Hebei	North	108	82	183	59.0%	44.8%
Jilin	North	11	11	166	6.6%	6.6%
Shanxi	North	27	27	139	19.4%	19.4%
Inner Mongolia	North	6	6	85	7.1%	7.1%
Shaanxi	Northwest	260	246	781	33.3%	31.5%
Xinjiang	Northwest	48	33	97	49.5%	34.0%
Sichuan	Southwest	190	174	758	25.1%	23.0%
Yunnan	Southwest	249	164	469	53.1%	35.0%
Hubei	Southwest	50	41	207	24.2%	19.8%
Guizhou	Southwest	24	1	137	17.5%	0.7%
Total	-	2416	1780	7518	32.1%	23.7%

**Table 4:** The number and percentage of the total/wild PRRSV PCR-positive batches in the 21 provinces.

Province	Production Region	Total PRRSV-Positive Batches	Wild PRRSV-Positive Batches	Total Batches	Percentage of Total PRRSV Batches	Percentage of Wild PRRSV Batches
Guangxi	Central–south	12	11	16	75.0%	68.8%
Guangdong	Central–south	32	27	42	76.2%	64.3%
Fujian	Central–south	33	26	44	75.0%	59.1%
Jiangxi	Central–south	27	19	34	79.4%	55.9%
Hunan	Central–south	3	3	9	33.3%	33.3%
Jiangsu	East	1	1	1	100.0%	100.0%
Henan	East	6	5	6	100.0%	83.3%
Shandong	East	31	24	32	96.9%	75.0%
Zhejiang	East	15	10	30	50.0%	33.3%
Hebei	North	16	15	17	94.1%	88.2%
Heilongjiang	North	19	17	23	82.6%	73.9%
Liaoning	North	10	10	19	52.6%	52.6%
Shanxi	North	5	5	10	50.0%	50.0%
Jilin	North	3	3	10	30.0%	30.0%
Inner Mongolia	North	1	1	4	25.0%	25.0%
Shaanxi	Northwest	17	17	18	94.4%	94.4%
Xinjiang	Northwest	7	5	8	87.5%	62.5%
Yunnan	Southwest	31	25	32	96.9%	78.1%
Hubei	Southwest	9	7	11	81.8%	63.6%
Sichuan	Southwest	28	22	59	47.5%	37.3%
Guizhou	Southwest	7	1	12	58.3%	8.3%
Total	-	313	254	437	71.6%	58.1%

## Data Availability

All the data underlying the findings described in this article are available within the article. For any data not included in the article, please contact the corresponding authors.

## Supplementary Materials

The following supporting information can be downloaded at: https://www.mdpi.com/article/10.3390/v16050774/s1, Table S1: The list of 390 ORF5 nucleotide sequences with corresponding information.; Table S2: Lineages distribution in each age class.
